# Genetic analysis of the vitamin D receptor gene in two epithelial cancers: melanoma and breast cancer case-control studies

**DOI:** 10.1186/1471-2407-8-385

**Published:** 2008-12-23

**Authors:** Eva Barroso, Lara P Fernandez, Roger L Milne, Guillermo Pita, Elena Sendagorta, Uxua Floristan, Marta Feito, Jose A Aviles, Manuel Martin-Gonzalez, Jose I Arias, Pilar Zamora, Monserrat Blanco, Pablo Lazaro, Javier Benitez, Gloria Ribas

**Affiliations:** 1 Human Genetics Group; Human Cancer Genetics Program, Spanish National Cancer Research Centre (CNIO), Madrid, Spain; 2 Genetic and Molecular Epidemiology Group; Human Cancer Genetics Program, CNIO, Madrid, Spain; 3 National Genotyping Centre (CeGen), Human Cancer Genetics Program, CNIO, Madrid, Spain; 4 Department of Dermatology, La Paz Hospital, Madrid, Spain; 5 Department of Dermatology, Gregorio Marañon Hospital, Madrid, Spain; 6 Department of Dermatology, Ramon y Cajal Hospital, Madrid, Spain; 7 Service of Surgery, Monte Naranco, Oviedo, Spain; 8 Department of Oncology, La Paz Hospital, Madrid, Spain

## Abstract

**Background:**

Vitamin D serum levels have been found to be related to sun exposure and diet, together with cell differentiation, growth control and consequently, cancer risk. Vitamin D receptor (*VDR*) genotypes may influence cancer risk; however, no epidemiological studies in sporadic breast cancer (BC) or malignant melanoma (MM) have been performed in a southern European population. In this study, the *VDR *gene has been evaluated in two epithelial cancers BC and MM.

**Methods:**

We have conducted an analysis in 549 consecutive and non-related sporadic BC cases and 556 controls, all from the Spanish population, and 283 MM cases and 245 controls. Genotyping analyses were carried out on four putatively functional SNPs within the *VDR *gene.

**Results:**

An association with the minor allele A of the non-synonymous SNP rs2228570 (rs10735810, *Fok*I, Met1Thr) was observed for BC, with an estimated odds ratio (OR) of 1.26 (95% CI = 1.02–1.57; p = 0.036). The synonymous variant rs731236 (*Taq*I) appeared to be associated with protection from BC (OR = 0.80, 95%CI = 0.64–0.99; p = 0.047). No statistically significant associations with MM were observed for any SNP. Nevertheless, sub-group analyses revealed an association between rs2228570 (*FokI*) and absence of childhood sunburns (OR = 0.65, p = 0.003), between the 3'utr SNP rs739837 (*Bgl*I) and fair skin (OR = 1.31, p = 0.048), and between the promoter SNP rs4516035 and the more aggressive tumour location in head-neck and trunk (OR = 1.54, p = 0.020).

**Conclusion:**

In summary, we observed associations between SNPs in the *VDR *gene and BC risk, and a comprehensive analysis using clinical and tumour characteristics as outcome variables has revealed potential associations with MM. These associations required confirmation in independent studies.

## Background

The vitamin D metabolite 1α,25-dihydroxivitamin D_3 _(1,25D, also known as calcitriol) is the biologically active form of vitamin D_3 _[1]. The concentration of vitamin D_3 _in natural foods is quite low, and the majority of vitamin D_3 _in individuals is from cholesterol metabolites in the skin upon exposure to ultraviolet (UV) radiation. 1,25D modulates the expression of specific genes in a tissue-specific manner by binding to the nuclear vitamin D receptor (*VDR*) and to specific DNA vitamin D response elements. The receptor and ligand induce a program of gene expression that contributes to the maintenance of the quiescent, differentiated phenotype. They are therefore able to regulate cellular proliferation, apoptosis and differentiation in many cell types [2].

Recent epidemiological studies have shown an association between low serum 1,25D levels and increased risk of breast, colorectal and prostate cancers. Furthermore, several studies have reported a possible link between polymorphic variants in the vitamin D receptor gene and increased susceptibility for primary and metastatic breast cancer, squamous cell carcinoma, colorectal cancer and prostate cancer [3-6]. Although the functional significance of these polymorphic variants remains unknown, there is strong evidence suggesting that they may have functional consequences in epithelial carcinogenesis and tumour progression [7,8]. *VDR *polymorphisms have been widely studied in Caucasian populations in relation to breast cancer (BC) [9-11] and malignant melanoma (MM) susceptibility [12,13], each finding different effects for SNPs, depending on the population analyzed and environmental factors acting upon them.

It is of general interest to study the most characterised variants in *VDR *in southern European countries, where sun exposure is typically higher than in northern European countries (maximum UV Index during the summer months = 9 in Spain versus 6.5 in Netherlands) [14,15] In this study, we investigated for the first time the role of polymorphisms in *VDR *in two epithelial cancers, sporadic BC and MM, in the Spanish population. Additionally, clinical and tumour phenotypic variables have been taken into account to better define the involvement of *VDR *in these pathologies.

## Methods

### Study Subjects, Data Collection and DNA Extraction

#### BC Study

The BC case-control study included a total of 549 consecutive and non-related sporadic BC cases and 556 control women. Cases were recruited from 1^st ^January 2002 to 31^st ^December 2006 from three Spanish public hospitals: 258 (47%) from Monte Naranco Hospital, in Oviedo; 155 (28%) from the Fundación Jiménez Díaz, and 136 (25%) from La Paz University Hospital, both in Madrid. Controls were unaffected Spanish women, recruited at three centres in Madrid: 455 (82%) from the Menopause Research Centre at the Instituto Palacios, 82 (15%) from the Fundación Jiménez Díaz, and 19 (3%) from the Madrid College of Lawyers. All cases and controls were women and controls were selected so that their age range was comparable to that of cases. We could not frequency match due to the larger numbers of cases.

Information about personal characteristics of cases and controls (age at diagnosis for cases or age at blood sample collection for controls, age at menarche, parity and menopausal status), and clinical and tumour characteristics for cases (metastasis at diagnosis, tumour grade, type and size, nodal involvement, and immunohistochemical markers), was either collected by the treating physician or extracted by review of medical records. This information is summarised in Additional file 1.

#### MM Study

The MM case-control study was based on 283 consecutive and non-related sporadic MM cases that were recruited from 1^st ^September 2004 to 15^th ^March 2008, at the Departments of Dermatology of three hospitals in Madrid: 147 (52%) from Gregorio Marañón General University Hospital, 54 (19%) from La Paz University Hospital and 82 (29%) from Ramón y Cajal University Hospital. A total of 245 cancer-free controls, frequency matched to cases by sex and age in ten-year categories, were recruited from the Madrid College of Lawyers (218 participants, 89%) and from Gregorio Marañón General University Hospital (27 participants, 11%).

A standardised questionnaire was used to collect information on pigmentation characteristics (eye colour, hair colour, skin colour, number of nevi, presence of solar lentigines), the presence of childhood sunburns, Fitzpatrick's classification of skin type, tumour location, Breslow index (deep index), and personal and family history of cancer, as described previously [16,17] (see Additional file 2). Fitzpatrick's classification of skin type was assessed for cases only, by review of medical records.

All participants in both studies were Caucasian and of Spanish origin. All subjects gave informed consent and the BC and MM studies were approved by the Ethics Committees of La Paz University Hospital and Gregorio Marañón General University Hospital, respectively.

Genomic DNA from cases and controls was extracted using the MagNA Pure LC Instrument according to the manufacturer's protocol as previously described [16,18,19].

### SNP selection

Three public databases were used to collect information about SNPs in *VDR*: NCBI , Ensembl , and HapMap . Four SNPs were considered for inclusion because they have been widely analysed in previous epidemiological studies. All had minor allele frequency (MAF) greater than or equal to 10%. Two are located on exons, one is in the putative promoter region and the other in the 3'utr region. The two coding SNPs selected have been reported to be associated with breast cancer, in previous studies [11,20,21].

### Genotyping assays

Genotyping was carried out using the TaqMan platform following the manufacturer's instructions. SNPs assays were designed using Applied Biosystems Assay-by-Design and Assay-on-Demand probes (Applied Biosystems, Foster City, CA, USA) (provided upon request). The genotype of each sample was automatically determined by measuring final allele-specific fluorescence in the ABI Prism 7900HT Detection System, using the SDS 2.1 software for allele discrimination (Applied Biosystems, Foster city, USA).

As a quality control measure, we included at least 2 sample duplicates and 1 non-template sample per 96-well plate. Genotypes were scored by two different personnel in the laboratory. We obtained a concordance rate of 100% for all four SNPs studied.

### Statistical Analysis

For all polymorphisms studied, Fisher's exact test was used both to test for deviations from Hardy-Weinberg equilibrium (HWE) among controls and to compare differences in the MAF distributions between cases and controls.

In order to assess associations between genotypes, haplotypes and cancer risk, several analyses were performed. Genotype-related odds ratios (ORs), their corresponding 95% confidence intervals (CIs) and associated p-values were estimated via unconditional logistic regression. This was done for each of heterozygotes and minor-allele homozygotes relative to common-allele homozygotes, as well as under an additive model, in the latter case estimating an effect per copy of the minor allele carried. Known or suspected risk factors for BC (age, number of live births, age at menarche, and menopause status) and MM (eye colour, hair colour, skin colour, number of nevi, lentigines, and childhood sunburns) were evaluated for potential confounding effects by including them in multivariate analyses.

Associations between *VDR *polymorphisms genotyped and various individual, clinical and tumour characteristics were assessed via logistic regression in order to determine their potential modifying effects on BC and MM risk. This was done for cases and controls pooled for each variable. Eye colour (blue/green versus brown), hair colour (blond/red versus brown/black), skin colour (fair versus brown), number of nevi (= 50 versus < 50), presence of lentigines (yes versus no) and childhood sunburn (yes versus no) were used as the outcome variables for MM.

Among BC cases only, the presence of metastastic disease at diagnosis (yes versus no), tumour histology (invasive versus in situ), tumour grade (grade > 1 versus grade 1), tumour size (> 2 cm versus = 2 cm), nodal involvement (yes versus no), estrogen receptor status (positive versus negative) and progesterone receptor status (positive versus negative), were used in the analysis. For MM cases-only analyses, the prior diagnosis of MM (yes versus no), phototype (I/II versus III/IV), tumour location (head/neck/trunk versus extremities) and tumour depth (T2/T3/T4 versus T0/T1) were considered as the outcome variables.

SPSS v11.0 was used to carry out these analyses. All p-values were two-sided and those less than 0.05 were considered statistically significant

## Results and Discussion

### Associations of *VDR *rs731276 and rs2228570 polymorphisms with cancer risk

Allelic frequencies for each SNP and the *p-value *for their comparison between cases and controls are presented in Table 1. We found no evidence of departure from Hardy-Weinberg equilibrium for any of the four SNPs genotyped (all p-values > 0.05). Results from univariate and multivariate genotype analysis are shown in Table 2.

**Table 1:** Allelic frequencies comparison between cases and controls in the four SNPs tested, in both BC and MM pathologies

				**BREAST CANCER**			**MELANOMA**			**HapMap**
	**Other names**	**SNP**	**Nucleotide**	**Cases (N = 549)**	**Controls (N = 556)**		**Cases (N = 283)**	**Controls (N = 245)**		**Caucasian**
**SNP ID**		**Location**	Change*	**MAF**	**MAF**	**p-value^++^**	**MAF**	**MAF**	**p-value^++^**	**MAF**
rs4516035		5' upstrem	T > C	0.41	0.39	0.27	0.44	0.40	0.28	0.45
rs2228570	*Fok*I. rs10735810	Met1Thr	G > A	0.37	0.34	0.08	0.31	0.33	0.33	0.44
rs731236	*Taq*I	Ile352	T > C	0.38	0.43	**0.028**	0.41	0.39	0.50	0.44
rs739837	*Bgl*I	3' utr	T > G	0.46	0.46	0.90	0.47	0.50	0.74	0.43

MAF, Minor Allele Frequency.

Statistically significant results (p < 0.05) indicated in bold.

* Correspondence of nomenclature of SNP alleles are as following: the FokI alleles G and A correspond to F and f, respectively; the TaqI alleles T and C

correspond to T and t, respectively; and BglI alleles T and G correspond to B and b, respectively.

^++^p-value, difference of MAF between cases and controls.

**Table 2:** Genotype frequencies comparison between cases and controls in the four SNPs tested, in both BC and MM pathologies

			**BREAST CANCER**				**MELANOMA**			
	**Statistical**	**Genotype**	**non-adjusted**		**adjusted^†^**		**non-adjusted**		**adjusted^‡^**	
**SNP ID**	**model**	**alleles**	OR* (95% CI)	**p-value**	OR* (95% CI)	**p-value**	OR* (95% CI)	**p-value**	OR* (95% CI)	**p-value**
rs4516035	Codominant	CT	1.20 (0.92–1.56)	0.19	1.15 (0.83–1.58)	0.40	1.08 (0.73–1.60)	0.69	1.17 (0.70–1.96)	0.54
		CC	1.16 (0.82–1.65)	0.41	0.97 (0.63–1.49)	0.88	1.51 (0.90–2.53)	0.12	1.79 (0.91–3.53)	0.09
	Per minor allele	C-	1.09 (0.92–1.29)	0.30	1.01 (0.82–1.25)	0.91	1.20 (0.94–1.55)	0.15	1.31 (0.94–1.81)	0.11
rs2228570	Codominant	GA	1.14 (0.88–1.47)	0.32	1.26 (0.89–1.66)	0.22	1.09 (0.75–1.57)	0.66	1.23 (0.76–2.01)	0.40
		AA	1.41 (0.96–2.08)	0.08	**1.65 (1.02–2.65)**	**0.041**	0.69 (0.38–1.25)	0.22	1.23 (0.55–2.73)	0.61
	Per minor allele	A-	1.17 (0.98–1.40)	0.08	**1.26 (1.02–1.57)**	**0.036**	0.91 (0.70–1.19)	0.49	1.15 (0.81–1.64)	0.43
rs731236	Codominant	CT	0.84 (0.64–1.09)	0.19	0.82 (0.59–1.13)	0.22	1.26 (0.85–1.87)	0.25	1.06 (0.63–1.78)	0.81
		CC	**0.70 (0.50–0.98)**	**0.040**	0.72 (0.48–1.09)	0.13	1.09 (0.64–1.84)	0.76	1.17 (0.57–2.39)	0.66
	Per minor allele	C-	**0.84 (0.71–0.99)**	**0.034**	0.85 (0.69–1.03)	0.10	1.08 (0.84–1.40)	0.55	1.08 (0.77–1.52)	0.66
rs739837	Codominant	TG	0.96 (0.72–1.27)	0.76	0.96 (0.69–1.35)	0.83	0.89 (0.59–1.36)	0.60	0.64 (0.36–1.13)	0.12
		GG	1.03 (0.74–1.44)	0.86	1.30 (0.87–1.96)	0.20	0.82 (0.50–1.36)	0.45	0.69 (0.35–1.38)	0.29
	Per minor allele	G-	1.01 (0.86–1.20)	0.87	1.13 (0.92–1.38)	0.25	0.91 (0.71–1.17)	0.45	0.83 (0.59–1.16)	0.28

*OR: Odds Ratio estimated under codominant and log-additive models; CI: Confidence Interval.

^†^Adjusted for age at diagnosis, number of live births, age at menarche and menopause status.

^‡^Adjusted for eye colour, hair colour, skin colour, number of nevi, lentigines, and childhood sunburn.

Statistically significant results (p < 0.05) indicated in bold.

We observed evidence of differences in minor allele frequency (MAF) between BC cases and controls for the synonymous change rs731236 (*Taq*I) (p = 0.028). The estimated OR per minor allele (C) in this SNP was 0.84 (95%CI 0.71–0.99, p = 0.034). This per-allele OR estimate was not substantially different in the multivariate analysis adjusting for age, number of live births, age at menarche, and menopause status (OR per allele = 0.85, 95% CI 0.69–1.03, p = 0.102). Regarding the SNP rs2228570 (*Fok*I) (Met1Thr, formerly known as rs10735810), weak evidence of differences in MAF between BC cases and controls was observed (p = 0.080). The estimated OR per minor allele in this SNP was 1.17 (95%CI 0.98–1.40, p = 0.081), whereas the per-allele OR estimated in the multivariate analysis adjusting for potential confounding factors was higher, and statistically significant (OR per allele = 1.26, 95%CI 1.02–1.57, p = 0.036).

In general, previous studies have found no evidence of association with BC for rs731236 (*Taq*I) and rs2228570 (*Fok*I) [9,11,22-27]. All of these studies had limited statistical power to detect a moderate association. Population stratification may be another explanation for the lack of consistency in results. However, two studies with marginal statistically significant results for rs731236 (*Taq*I) reported contradictory results [11,24], whereas studies using larger sample sizes from Caucasian populations have shown risk effect of rs2228570 (*Fok*I) consistent with that detected in the present study [20,21]. An association with rs2228570 (*Fok*I) was observed after adjustment for established risk factors including those used in the present study. This difference may be due to the tight relationship between *VDR *protein function and the hormonal aspect of BC aetiology such as menarche, parity and menopause [28,29].

In the case of MM, we did not observe any evidence of association with rs731236 (*Taq*I) (OR per allele = 1.08, 95%CI = 0.84–1.40, p = 0.55) or rs2228570 (*Fok*I) (OR per allele = 0.91 95%CI = 0.70–1.19, p = 0.49). These results are consistent with the findings of smaller sample size studies [13,30,31] although other studies reported a protective tendency of rs731236 (*Taq*I) [12,32]. Only two studies reported an association of rs2228570 (*Fok*I) and MM risk in North-European populations, one of them being a larger sample size study [12,32].

The rs731236 (*Taq*I) SNP is in linkage disequilibrium with other polymorphisms in the 3' extreme of the gene in Caucasian populations. Functional studies of these polymorphisms have evaluated their putative implication in the regulation of transcription, translation or RNA processing, but no consistent results were obtained [33]. However, functional studies of rs2228570 (*Fok*I) have suggested a loss of the reported *VDR *benefits induced by the minor allele, due to its location in the first codon of the protein (Met1Thr). That is, the minor A allele appears to be associated with the use of an alternate start codon, which triggers a longer and less potent transcriptional activator protein form [7], which is consistent with it being associated with increased risk of BC. We did not observe evidence of association for any other SNP in BC or MM.

### Associations of *VDR *polymorphisms with personal, clinical and tumoral characteristics

We assessed whether *VDR *SNPs were associated with various clinical and phenotypic characteristics using cases and controls combined. We tested for associations with tumour characteristics among cases only for each disease. Results are summarised in Table 3. The rare allele in the non-synonymous SNP rs2228570 (*Taq*I) appeared to be strongly associated with the absence of childhood sunburns (OR per allele = 0.65, 95% CI 0.49–0.86, p = 0.003), and this was maintained among controls only (OR per allele = 0.63). There was also weak evidence that it is associated with a prior diagnosis of MM in MM patients (p = 0.060). We also observed marginally significant associations for the 3'utr SNP rs739837 (*Bgl*I) with fair skin colour (p = 0.048) and with Fitzpatrick's phototype I/II (0.070). Finally, the *VDR *promoter SNP rs4516035 was associated with tumours located in the head-neck or trunk (p = 0.020). No other associations were observed for BC or MM. Although we did not detect a significant effect of *VDR *SNPs directly on MM, the associations identified with MM phenotypic characteristics suggest that *VDR *SNPs may modulate MM susceptibility.

**Table 3:** Personal, clinical and tumoral phenotypic characteristics comparison from both BC and MM pathologies in the four SNPs tested

**Tumour**		**rs4516035**		**rs2228570**		**rs731236**		**rs739837**	
**Type**	**Characteristic**	OR* (95% CI)	**p-value**	OR* (95% CI)	**p-value**	OR* (95% CI)	**p-value**	OR* (95% CI)	**p-value**
**BREAST CANCER**									

	Metastasis^‡^	0.53 (0.25–1.11)	0.09	1.00 (0.51–1.96)	0.99	1.20 (0.63–2.27)	0.58	1.11 (0.57–2.17)	0.75
	Tumor histology (Invasive)^‡^	0.87 (0.57–1.32)	0.51	0.83 (0.54–1.28)	0.40	1.10 (0.72–1.69)	0.66	0.75 (0.49–1.15)	0.19
	Tumor grade (Grade > 1)^‡^	0.93 (0.67–1.30)	0.68	1.19 (0.84–1.68)	0.33	1.22 (0.87–1.70)	0.26	0.76 (0.54–1.05)	0.09
	Tumor size (> 2 cm)^‡^	1.02 (0.77–1.35)	0.92	0.98 (0.73–1.30)	0.88	1.14 (0.86–1.51)	0.37	0.81 (0.61–1.08)	0.15
	Nodal involvement^‡^	0.93 (0.70–1.23)	0.61	0.95 (0.71–1.27)	0.72	0.94 (0.71–1.25)	0.69	1.09 (0.82–1.45)	0.54
	ER positive^‡^	0.96 (0.66–1.41)	0.85	1.24 (0.84–1.84)	0.28	1.31 (0.90–1.90)	0.16	1.02 (0.71–1.46)	0.91
	PR positive^‡^	0.92 (0.67–1.26)	0.60	1.12 (0.80–1.55)	0.51	0.91 (0.67–1.24)	0.56	1.12 (0.83–1.51)	0.48

**MELANOMA**									

	Light Eye Colour^+^	1.03 (0.79–1.34)	0.82	0.91 (0.69–1.21)	0.52	0.92 (0.70–1.21)	0.56	1.06 (0.81–1.39)	0.64
	Blond/Red Hair Colour^+^	0.99 (0.71–1.40)	0.99	0.78 (0.55–1.13)	0.19	1.02 (0.73–1.44)	0.89	1.45 (0.89–1.74)	0.20
	Fair Skin Colour^+^	0.97 (0.75–1.26)	0.83	1.08 (0.82–1.42)	0.57	0.86 (0.66–1.12)	0.25	**1.31 (1.00–1.70)**	**0.048**
	N° of Nevi = 50^+^	0.97 (0.66–1.40)	0.85	1.00 (0.67–1.49)	0.99	1.06 (0.72–1.54)	0.77	1.34 (0.90–1.97)	0.15
	Presence of Lentigines^+^	1.03 (0.79–1.34)	0.84	0.79 (0.59–1.05)	0.11	1.20 (0.91–1.58)	0.21	0.90 (0.69–1.18)	0.45
	Presence of Childhood Sunburns^+^	1.12 (0.87–1.45)	0.39	**0.65 (0.49–0.86)**	**0.003**	1.10 (0.84–1.43)	0.49	0.91 (0.70–1.19)	0.50
	Other MM^‡^	1.26 (0.52–3.03)	0.61	2.43 (0.95–6.19)	0.060	1.83 (0.73–4.56)	0.19	0.70 (0.26–1.85)	0.47
	Fitzpatrick's photoype I/II^‡^	0.74 (0.51–1.09)	0.13	0.85 (0.63–1.46)	0.89	0.91 (0.62–1.34)	0.63	1.43 (0.97–2.12)	0.070
	Tumor Location (Head/Neck/Trunk)^‡^	**1.54 (1.08–2.20)**	**0.020**	1.16 (0.79–1.72)	0.45	1.16 (0.81–1.65)	0.42	1.17 (0.82–1.67)	0.38
	Breslow Index (T2/T3/T4)^‡^	1.04 (0.72–1.51)	0.82	1.20 (0.80–1.79)	0.38	0.84 (0.57–1.24)	0.38	1.01 (0.69–1.47)	0.96

*OR: Odds Ratio per minor allele; CI: Confidence Interval, unadjusted p-values.

^+^Cases and controls pooled for each variable.

^‡^Cases only considered.

Statistically significant results (p < 0.05) indicated in bold.

### Further considerations

The strength of our study is the ability to control for the many established risk factors for BC and MM, although we recognize that there was potential for misclassification of phenotypic characteristics due to the subjective nature of the phenotypic attributes considered. Controls participated on a volunteer basis which may have introduced some selection bias. However, the fact that they were frequency matched to cases on age and sex for melanoma and that breast cancer controls were selected so that their age range was comparable to that of cases and that the variable of primary interest was genetic would have kept such bias to a minimum. It should be noted that, the sample size of both studies was relatively limited and so associations can not be ruled out for rs731236 (*Taq*I), and rs2228570 (*Fok*I), particularly in MM. The association of rs731236 (*Taq*I) and BC, was not statistically significant under a multivariate model. However, the estimated relative risk did not change substantially, indicating that the increase in p-value was due to the reduced sample size (with available covariate data), rather than due to confounding. Finally, conclusions are based on nominal p-values at 5% statistical significance and therefore require replication in independent studies.

## Conclusion

BC and MM tumour pathologies may be influenced by the effect of variation in vitamin D intake through diet and sun exposure, together with *VDR *polymorphisms. Therefore, in a sunny region such as Spain, the effect of *VDR *polymorphisms on cancer risk may be more apparent than in other Caucasian populations. The results obtained in this study add to the evidence for a role of *VDR *as an important mediator in the development of cancer. We found evidence of association with BC for the non-synonymous variant rs2228570 (*FokI*), and for the synonymous SNP rs731236 *(TaqI)*. These findings require replication in large samples and the role of these variants needs to be clarified by functional studies. We have also reported several associations among *VDR *SNPs and phenotypic risk factors that influence MM susceptibility, indicating their potential effect in disease development. The characterization of these and other polymorphisms in the VDR gene may help to better understand the aetiology and development of cancer, and to define risk groups to better target prevention strategies.

## Abbreviations

VDR: Vitamin D receptor; BC: Breast cancer; MM: Malignant melanoma; SNP: Single nucleotide polymorphism; OR: Odds ratio; UV: Ultraviolet; DNA: Deoxyribonucleic acid; MAF: Minor alelle frequency; HWE: Hardy-Weinberg equilibrium; CIs: Confidence Intervals

## Competing interests

The authors declare that they have no competing interests.

## Authors' contributions

EB and LPF participated in the design of the study, acquisition of data, genotyping, analysis and interpretation of data, performed the statistical analysis and drafted the manuscript. RLM performed the interpretation of data, the statistical analysis and drafted the manuscript. GP has been involved in the acquisition, designing sample data-base used for the study and interpretation of data. ES, UF, MF, JAA, MM, JIA, PZ, MB and PL participated in patient enrolment and patient phenotypic classification and the acquisition of clinical data. JB have been involved in revising the manuscript and in given final approval of the version to be published. GR participated in the design of the study, analysis and interpretation of data, revising the manuscript and in given final approval of the version to be published.

The manuscript has been seen and approved by all listed authors. E Barroso and LP Fernandez contributed equally to this work.

## Pre-publication history

The pre-publication history for this paper can be accessed here:



## Supplementary Material

Additional file 1 **Personal, clinical and tumoral phenotypic characteristics in cases and controls in BC**. The data provided represent the personal, clinical and tumoral phenotypic characterization of BC samples used in the study. Click here for file

Additional file 2 **Personal, clinical and tumoral phenotypic characteristics in cases and controls in MM.** The data provided represent the personal, clinical and tumoral phenotypic characterization of MM samples used in the study. Click here for file
