# Supplementary material for: Genetic analysis of the vitamin D receptor gene in two epithelial cancers: melanoma and breast cancer case-control studies
Source: BMC Cancer. 2008 Dec 23;8:385. doi: 10.1186/1471-2407-8-385 (PMC2639605; doi:10.1186/1471-2407-8-385)
Supplement: Additional file 1 — Personal, clinical and tumoral phenotypic characteristics in cases and controls in BC. The data provided represent the personal, clinical and tumoral phenotypic characterization of BC samples used in the study. [file 1471-2407-8-385-S1.doc]

| **Additional file 1: Personal, clinical and tumoral phenotypic** | | | |
| --- | --- | --- | --- |
| **characteristics in cases and controls in BC** | |  |  |
|  | **Controls (N=556)** | **Cases (N=549)** | **p-value*** |
| **Characteristic** | **n (%)** | **n (%)** |  |
| **Age (years)** |  |  | 2.51 x 10^-7^ |
| Median (range) | 53 (23-82) | 58 (23-89) |  |
| < Median (53y) | 269 (48.38) | 171 (31.15) |  |
| ≥ Median (53y) | 287 (51.62) | 349 (63.57) |  |
| Unknown | - | 29 (5.28) |  |
| **Age at menarche (years)** |  |  | 0.16 |
| Median (range) | 13 (9-18) | 13 (9-19) |  |
| < Median (13y) | 162 (29.14) | 141 (25.68) |  |
| ≥ Median (13y) | 228 (51.80) | 309 (56.28) |  |
| Unknown | 106 (19.06) | 99 (18.03) |  |
| **Parity (number of life births)** | |  | 2.4 x 10^-3^ |
| Median (range) | 2.13 (1.25) | 1.92 (1.44) |  |
| Nulliparous (0 life birth) | 55 (9.89) | 78 (14.21) |  |
| Parous (≥ 1 life birth) | 395 (71.04) | 313 (57.01) |  |
| Unknown | 106 (19.06) | 158 (28.78) |  |
| **Menopause status** |  |  | 2.4 x 10^-9^ |
| Premenopause | 224 (40.29) | 121 (22.04) |  |
| Postmenopause | 332 (59.71) | 399 (72.68) |  |
| Unknown | - | 29 (5.28) |  |
| **Metastasis at diagnosis** |  |  | - |
| M0 | - | 444 (80.87) |  |
| M1 | - | 20 (3.64) |  |
| Unknown | - | 85 (15.48) |  |
| **Tumour histology** |  |  | - |
| In situ | - | 51 (9.29) |  |
| Invasive | - | 373 (67.94) |  |
| Others | - | 26 (4.74) |  |
| Unknown | - | 99 (18.03) |  |
| **Tumour grade** |  |  | - |
| Grade 1 | - | 99 (18.03) |  |
| Grade > 1 | - | 270 (49.18) |  |
| Unknown | - | 180 (32.79) |  |
| **Tumour size** |  |  | - |
| ≤ 2cm | - | 316 (57.56) |  |
| > 2cm | - | 147 (26.78) |  |
| Unknown | - | 86 (15.66) |  |
| **Nodal involvement** |  |  | - |
| Negative | - | 284 (51.73) |  |
| Positive | - | 156 (28.42) |  |
| Unknown | - | 109 (19.85) |  |
| **ER** |  |  | - |
| Negative | - | 79 (14.39) |  |
| Positive | - | 244 (44.44) |  |
| Unknown | - | 226 (41.17) |  |
| **PR** |  |  | - |
| Negative | - | 142 (25.87) |  |
| Positive | - | 202 (36.79) |  |
| Unknown | - | 205 (37.34) |  |
| * Fisher's exact test. P value excluding unknown values. | | |  |
| SD, standard deviation. |  |  |  |
| Statistically significant results (p<0.05) indicated in bold | | |  |
