# Supplementary material for: Genetic analysis of the vitamin D receptor gene in two epithelial cancers: melanoma and breast cancer case-control studies
Source: BMC Cancer. 2008 Dec 23;8:385. doi: 10.1186/1471-2407-8-385 (PMC2639605; doi:10.1186/1471-2407-8-385)
Supplement: Additional file 2 — Personal, clinical and tumoral phenotypic characteristics in cases and controls in MM. The data provided represent the personal, clinical and tumoral phenotypic characterization of MM samples used in the study. [file 1471-2407-8-385-S2.doc]

| **Additional File 2: Personal, clinical and tumoral phenotypic** | | | |
| --- | --- | --- | --- |
| **characteristics in cases and controls in MM** | | |  |
|  | **Cases (N=283)** | **Controls (N=245)** | **p-value*** |
| **Characteristic** | **n (%)** | **n (%)** |  |
| **Age at diagnosis (years)** | |  | 0.31 |
| Mean (SD) | 47.17 (21.93) | 53.35 (15.67) |  |
| < Mean | 139 (49.12) | 136 (55.51) |  |
| ≥ Mean | 132 (46.64) | 108 (44.08) |  |
| Unknown | 1 (0.41) | 12 (4.24) |  |
| **Sex** |  |  | 0.38 |
| Male | 129 (45.58) | 123 (50.20) |  |
| Female | 153 (54.06) | 122 (49.80) |  |
| Unknown | 1 (0.35) | - |  |
| **Eye Colour** |  |  | **8.6 x 10^-5^** |
| Light Eye Colour | 113 (39.93) | 59 (24.08) |  |
| Dark Eye Colour | 165 (58.30) | 183 (74.69) |  |
| Unknown | 5 (1.77) | 3 (1.22) |  |
| **Hair Colour** |  |  | **1.3 x 10^-8^** |
| Blond/Red | 72 (25.44) | 18 (0.41) |  |
| Brown/Black | 206 (72.79) | 226 (92.24) |  |
| Unknown | 5 (1.77) | 1 (0.41) |  |
| **Skin Colour** |  |  | 0.27 |
| Fair Skin Colour | 178 (62.90) | 141 (57.55) |  |
| Dark Skin Colour | 101 (35.69) | 98 (40.00) |  |
| Unknown | 4 (1.41) | 6 (2.45) |  |
| **Nº of Nevi** |  |  | 0.23 |
| <50 | 234 (82.69) | 180 (73.47) |  |
| ≥50 | 44 (15.55) | 24 (9.80) |  |
| Unknown | 5 (1.77) | 41 (16.73) |  |
| **Lentigines** |  |  | **7.8 x 10^-7^** |
| No | 74 (26.15) | 104 (42.45) |  |
| Yes | 202 (71.38) | 110 (44.90) |  |
| Unknown | 7 (2.47) | 31 (12.65) |  |
| **Childhood sunburn** | |  | **1.1 x 10^-14^** |
| No | 87 (30.74) | 171 (69.80) |  |
| Yes | 187 (66.08) | 42 (17.14) |  |
| Unknown | 9 (3.18) | 32 (13.06) |  |
| **Other MM** |  |  | - |
| No | 261 (92.20) | - |  |
| Yes | 10 (3.50) | - |  |
| Unknown | 12 (4.20) | - |  |
| **Breslow thickness** |  |  | - |
| T0/T1 | 164 (57.95) | - |  |
| T2T3/T4 | 89 (31.45) | - |  |
| Unknown | 30 (10.60) | - |  |
| **Tumor histology** | |  | - |
| Superficial Spreading | 184 (65.02) | - |  |
| Lentigo Maligna | 15 (5.30) | - |  |
| Nodular | 31 (10.95) | - |  |
| Others | 6 ((2.12) | - |  |
| Unknown | 37 (13.07) | - |  |
| **Tumor Location** |  |  | - |
| Head/Neck/Trunk | 170 (60.07) | - |  |
| Extremities | 108 (38.16) | - |  |
| Unknown | 5 (1.77) | - |  |
| **Fitzpatrick phototype** | |  | - |
| I-II | 128 (45.23) | - |  |
| III-IV | 104 (36.75) | - |  |
| Unknown | 51 (18.02) | - |  |
| * Fisher's exact test. P value excluding unknown values. | | |  |
| SD. standard deviation | |  |  |
| Statistically significant results (p<0.05) indicated in bold | | |  |
